# Description of a Skeleton of the Mastodon Giganteus of North America

**Published:** 1854-10

**Authors:** 


Art. V.—*Description of a Skeleton of the Mastodon Giganteus of North America.* By. John C. Warren, M. D., late Professor of Anatomy in the University of Cambridge; President of the Boston Society of Natural History; Member of the American Philosophical Society, of the American Academy of Arts and Sciences, of the Philadelphia Academy of Natural Sciences, of the Academy of Naples, and the Medical Society Florence; Honorary member of the Medico-Chirurgical Society of London; Corresponding member of the Academy of Medicine at Paris, etc., etc. Boston: 1852. 4to., pp. 219

*Remarks on some Fossil Impressions in the Sandstone rocks of Connecticut River.* By John C. Warren, M. D., etc., etc. Boston: Ticknor & Fields. 1854. 8vo. pp. 54.

The author of these works has long stood prominent among the distinguished medical men of America. Dr. Warren has devoted a long life to the unwearied cultivation and advancement of his profession, in the midst of a laborious practice finding time to enrich its literature by a succession of works marked by sound judgment, learning, and originality. An able and successful surgeon and teacher, he has been a diligent cultivator of comparative anatomy. More than thirty years ago he published a treatise on the *Nervous Systems of Men and Animals*, evincing accurate knowledge and deep research; and now, at an age when most men are content to seek repose, we find him following it up with a work on a kindred subject, which, if he had given nothing else to the world, would have placed him in the foremost rank of American naturalists.

Dr. Warren has been, for a number of years, the fortunate possessor of one of the most perfect skeletons of the Mastodon to be found in any cabinet, and to this circumstance we are, in part, indebted for this magnificent work. We rejoice that these interesting remains fell into the hands of one so competent, in every respect, to record the history of this monarch of our primeval forests. The volume is one of exceeding beauty and richness, and for the elegant form in which it appears we are wholly indebted to the munificence of its learned author.

No one, we should think, can be entirely indifferent to the history of this wonderful race of extinct beings, which at one period so abounded on our continent but long since has passed away, without leaving behind among the mammalian family one worthy representative. Their remains have been found scattered, in greater or less numbers, over a large portion of the United States and Canada. Herds of these huge creatures seem to have congregated, at times, about the saline springs of Kentucky, in the neighborhood of some of which vast collections of their bones have been discovered. The range of the Mastodon giganteus extended from the Connecticut river to the Willamet, in Oregon, where their skeletons have lately been found by an exploring expedition. It is a remarkable fact, recorded by Dr. Warren, that scarcely any of their remains have been known east of the Hudson river, and none east of the Connecticut, which cannot be explained satisfactorily by the severity of the climate, for Canada, still further north, presents a number of their relics. Some peculiarity in the soil, or productions of this section of the country, he thinks, may have been uncongenial to these animals; or possibly, he conjectures, New England may have been separated from the continent, in the time of the mastodon, by an arm of the sea too wide to have been readily crossed by the race.

It is a curious question what physical circumstances led to the extermination of this gigantic race. That they flourished at a period anterior to the human era there can be no doubt, since there is no tradition among any nation of men of their existence. They had become extinct before the present tribes of Indians took possession of America. By some it has been conjectured that they perished during the age in which the *drift* was spread over our continent, probably submerged under the great flood which brought down that deposit. But to this theory there is the grave objection, that their remains are found where there is no evidence of diluvial action. The drift ceases about the fortieth degree of north latitude, while bones of the mastodon have often been exhumed as far south as Alabama and Mississippi.

The fine specimen in the collection of Dr. Warren was found in the State of New York, near Newburgh, on the Hudson River, in the summer of 1845. Some laborers, engaged in removing the earth from the bottom of a pond to fertilize the fields of Mr. N. Brewster, struck their spade against some solid body, which on examination proved to be the head of the mastodon. "This, being uncovered, disclosed to the eyes of the spectators the full extent of the cranium, which was four feet in length. The lower jaw was distorted a little towards the left side. The bones of the spine, tail, pelvis, and ribs were successively found, for the most part in their natural relation to each other. The anterior extremities were extended under and in front of the head, as if the animal had stretched out its arms in a forward direction to extricate itself from a morass, into which it had sunk. The posterior extremities were extended forward under the body. The tusks lay with their convexities outwards, their anterior extremities opposed to each other nearly meeting." There, almost on the surface of the earth, scarcely covered by the soil and a few feet of water, they had remained undisturbed for unknown ages. The bones were well preserved, not black as are most of these remains, but of a brown color, like those of a recent human skeleton which had been a considerable time in use.

The bones of this creature when first discovered were supposed to pertain to the primitive elephant, but the mammillated character of the tooth soon convinced the great Cuvier, that they belonged to an animal of a different genus. In point of size the mastodon stood at the head of all the terrestrial mammalia. Dr. WARREN, in his faithful description of its osteology, says: "Language is insufficient to give an idea of the grandeur of the skeleton as a whole. Standing as it does in the midst of those of various large animals,—the horse, the cow, &c., and towering above them, its massive limbs make them sink into insignificence. Even the elephant, although nearly as tall, has a frame which might be called delicate when compared with that of the mastodon."

In the second of the works noticed at the head of this article, Dr. WARREN gives a description, which will be read with interest by geologists, of the fossil impressions in the sand-stone rocks of Connecticut river, preceded by an account of the *Epyornis*, or great Bird of Madagascar. The impressions to which Dr. WARREN refers are supposed to be, principally, the tracks of huge extinct birds, that in by-gone ages frequented the shores of that ancient sea. They constitute a study in which paleontologists have engaged with great zeal, under the names of Ichnology and Ornithicnites. To a geologist of our own country, Dr. HITCHCOCK, President of Amherst College, the scientific world is indebted for most of the facts upon which the newly created science of Ornithicnites rests. Besides the impressions of birds, the tracks of frogs, lizards, turtles, fishes, mollusca, crustacea, worms, and zoophytes have been perpetuated in the rocks of many countries, and the discovery of the former has served to carry back the creation of birds to an era much earlier than they were once believed to have existed.

But we must not run into details on this fascinating subject, which many of our readers, we fear, would deem rather foreign from medicine. In concluding this brief notice of these works, we cannot refrain from expressing our thanks to Dr. WARREN for the favor which their production has conferred upon American science.

